# p53 Inhibition in Pancreatic Progenitors Enhances the Differentiation of Human Pluripotent Stem Cells into Pancreatic β-Cells

**DOI:** 10.1007/s12015-023-10509-1

**Published:** 2023-01-28

**Authors:** Idil I. Aigha, Essam M. Abdelalim

**Affiliations:** 1 Diabetes Research Center (DRC), Qatar Biomedical Research Institute (QBRI), Qatar Foundation (QF), Hamad Bin Khalifa University (HBKU), PO Box 34110, Doha, Qatar; 2 College of Health and Life Sciences, Qatar Foundation (QF), Hamad Bin Khalifa University (HBKU), Doha, Qatar

**Keywords:** hESCs, Differentiation protocol, NKX6.1, Insulin, Islets, Monohormonal β-cells

## Abstract

The multipotent pancreatic progenitor cells (MPCs) co-expressing the transcription factors, PDX1 and NKX6.1, are the source of functional pancreatic β-cells. The aim of this study was to examine the effect of p53 inhibition in MPCs on the generation of PDX1^+^/NKX6.1^+^ MPCs and pancreatic β-cell generation. Human embryonic stem cells (hESCs) were differentiated into MPCs and β-cells. hESC-MPCs (stage 4) were treated with different concentrations of p53 inhibitors, and their effect was evaluated using different approaches. NKX6.1 was overexpressed during MPCs specification. Inhibition of p53 using pifithrin-μ (PFT-μ) at the MPC stage resulted in a significant increase in the number of PDX1^+^/NKX6.1^+^ cells and a reduction in the number of CHGA^+^/NKX6.1^−^ cells. Further differentiation of MPCs treated with PFT-μ into pancreatic β-cells showed that PFT-μ treatment did not significantly change the number of C-Peptide+ cells; however, the number of C-PEP+ cells co-expressing glucagon (polyhormonal) was significantly reduced in the PFT-μ treated cells. Interestingly, overexpression of NKX6.1 in hESC-MPCs enhanced the expression of key MPC genes and dramatically suppressed p53 expression. Our findings demonstrated that the p53 inhibition during stage 4 of differentiation enhanced MPC generation, prevented premature endocrine induction and favored the differentiation into monohormonal β-cells. These findings suggest that adding a p53 inhibitor to the differentiation media can significantly enhance the generation of monohormonal β-cells.

**Graphical Abstract:**

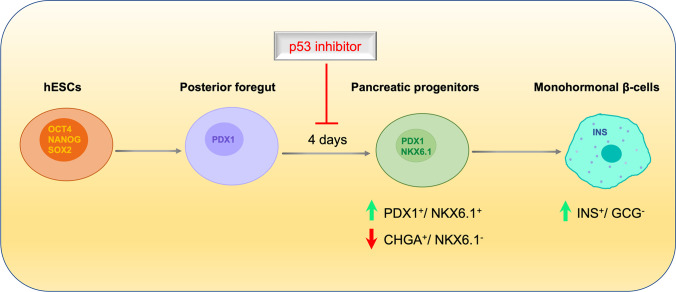

**Supplementary Information:**

The online version contains supplementary material available at 10.1007/s12015-023-10509-1.

## Introduction

The loss of functional pancreatic β-cells mass marks the onset and development of type 1 and type 2 diabetes (T1D and T2D). T1D is characterized by the autoimmune destruction of pancreatic β-cells that regulate blood glucose, whereas T2D progresses due to the gradual loss of functional β-cells associated with insulin resistance in the insulin target tissues [1, 2]. Several studies have explored cell replacement therapies as an alternative strategy for the treatment of diabetes. These therapeutic interventions that are based on replenishing functional β-cells mass, can possibly resolve the fluctuations in glucose homeostasis and the life-long dependency on insulin therapy. This was achieved through transplantation of whole pancreas or isolated cadaveric islets [3–5]. However, despite the promising clinical outcome of the islet transplantation, the feasibility of this approach is limited by the inadequate supply and quality of these islets [6].

Human pluripotent stem cell (hPSC)-derived pancreatic β-cells could potentially serve as an alternative source for cellular therapy of diabetes. Several studies reported the generation of insulin expressing hPSC-β cells using stepwise differentiation protocols (reviewed in [7–9]) that employed a cocktail of signaling cues, which are adapted from developmental signals involved during in vivo β-cell development [10, 11]. However, one of the major drawbacks in the utilization of the generated hPSC-β cells is the heterogeneity of the generated cells with the lack of sufficient pure populations of functional insulin secreting hPSC-β [12]. Differentiation protocols aiming to generate functional β-cells in vitro using hPSC-β, have demonstrated that the expression of NKX6.1 in PDX1^+^ multipotent pancreatic progenitor cells (MPCs) is a prerequisite for functional hPSC-β-cells. On the other hand, progenitors lacking NKX6.1 expression (PDX1^+^/NKX6.1^−^) develop into polyhormonal cells and fail to respond to glucose [13–17]. Several studies have highlighted the indistinguishable role of NKX6.1 during the pancreas development and, in generating glucose responsive-insulin producing β-cells [18].

p53 is a tumor suppressor, regulating several biological functions such as apoptosis, DNA repair, and cell cycle. Several evidence demonstrated that p53 is involved in other aspects of human physiology including the processes of organogenesis and differentiation [19]. However, the role of p53 during pancreatic β-cell differentiation is still not clear. In this present study, our aim was to examine the effect of the chemical inhibition of p53 on the generation of MPCs co-expressing PDX1+/NKX6.1+ cells during pancreatic β-cell differentiation. Our study showed that inhibition of p53 during MPC differentiation enhances the generation of MPCs co-expressing PDX1 and NKX6.1 and favors the pancreatic β-cell lineage. Also, we report that NKX6.1-mediated p53 inhibition, is a novel mechanism involved in controlling the generation of functional monohormonal β- cells from MPCs.

## Materials and Methods

### Culture of Human Pluripotent Stem Cells

hESC line (HUES8) was obtained from Harvard Stem Cell Institute (Cambridge, Madison, USA) and H1-hESC line was obtained from WiCell Research Institute (Maddison, USA). Both cell lines were cultured with mTesR1 medium (Stem Cell Technologies, Canada) and maintained in 5% CO2 at 37 °C on Matrigel-coated (Corning, USA) cell culture plates. Differentiation was started after 48–72 hr. after passaging with a confluency of ~70%. Cells were dissociated with the use of ReLeSR (Stem Cell Technologies, Canada) for 2–4 min or until detachment of the colony borders, and then the cells were collected and resuspended in mTesR1 medium supplemented with 10 μM Y-27632 (Rock inhibitor) (Stemgent, USA) for first 24 h of passaging.

### Differentiation of hESCs into Pancreatic Progenitors and β Cells

**Fig. 1:**
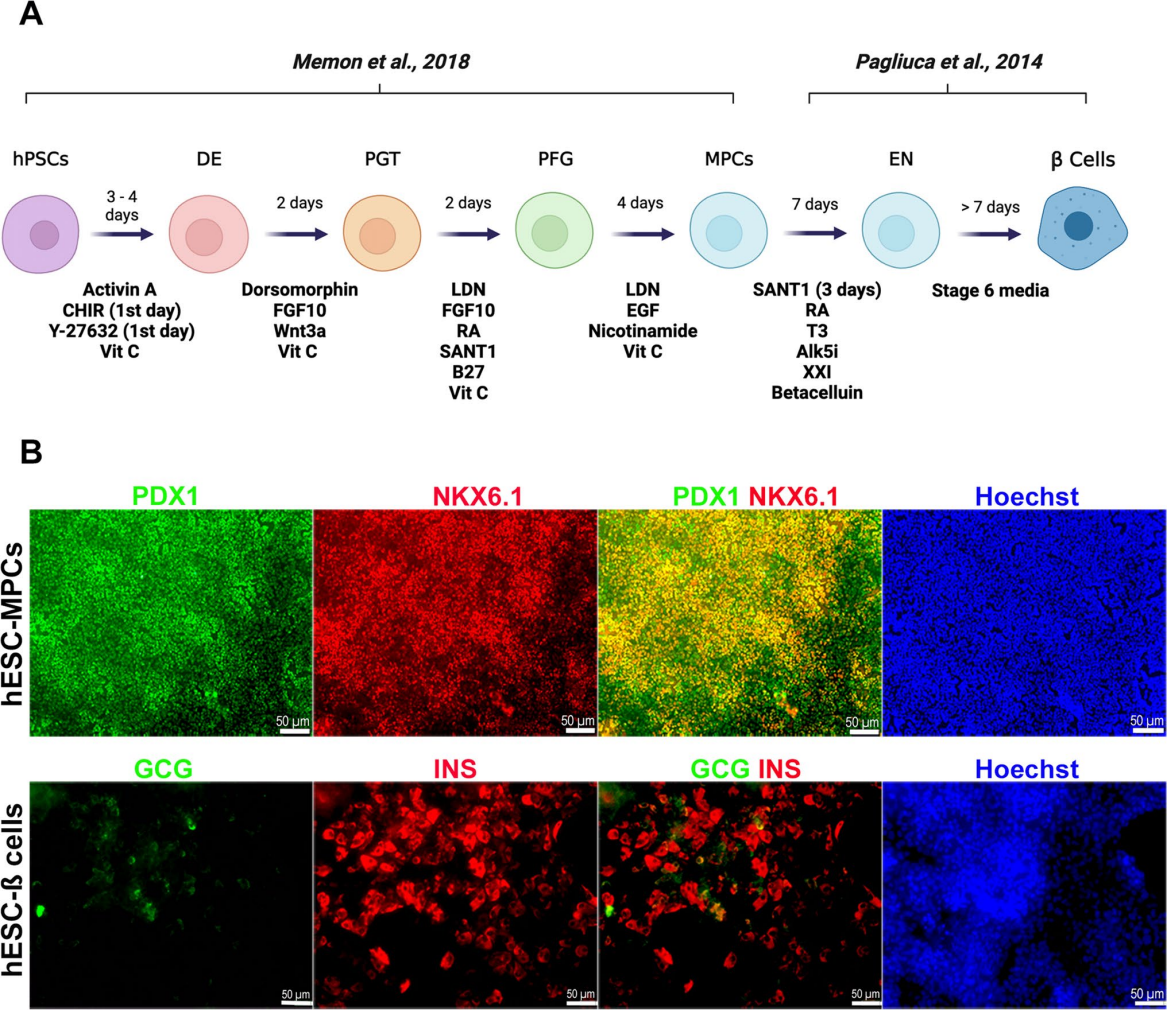
Differentiation of hESCs into pancreatic progenitors and pancreatic β-cells. (**A**) Schematic diagram showing the differentiation protocols used to generate multipotent pancreatic progenitor cells (MPCs) and pancreatic β-cells. (**B**) Immunofluorescence analysis for the co-expression of PDX1 and NKX6.1 and glucagon (GCG) and insulin (INS) in hESC-derived MPCs and hESC-derived pancreatic β-cells. Scale bars = 50 μm

Differentiation was initiated when hPSCs reached 80% confluence. We used two protocols to generate MPCs as described in Supplementary Table 1. We used our established protocol of Memon et al. [20–22] to generate MPCs (pancreatic progenitors). For further differentiation of MPCs into pancreatic β-cells, updated version (v8) of Pagliuca et al., was used [16]. mTeSR1 media was replaced with MCDB131 and/or DMEM/F12 (Thermo Fisher Scientific) supplemented with the stage-specific cytokines and growth factors (the detailed protocol and cytokines were described in Fig. 1A, Supplementary Table 1). p53 inhibitors, pifithrin (PFT)-α and PFT-μ (Sigma, USA), were added at two different concentrations (10 μM and 20 μM) for 4 days during stage 4 of differentiation. During stage 5, 20 μM PFT-μ were added daily.

### Reverse Transcription-Polymerase Chain Reaction (RT-PCR) and Real-Time PCR (qPCR)

Total RNA was isolated from the differentiated cells using Direct-zol RNA Purification kit (Zymo Research, USA) following the manufacturer’s recommendations. Superscript™ IV, first-strand synthesis system kit (Thermo Fisher Scientific) was used to synthesize the cDNAs. PCR-Master mix (2×) from (Thermo Fisher Scientific) was used for conventional PCR. The list of primers used are listed in Supplementary Table 2. The RT-PCR products were analyzed by agarose gel electrophoresis. For quantitative RT-PCR (qRT-PCR), SYBR Green-based detection system (GoTaq qPCR Master Mix, Wisconsin, USA) was used to quantify the expression level of mRNA levels. Relative quantification was performed using the comparative ΔΔCT method for each transcript. The experiment was performed using QuantStudio 7 Flex system (Applied Biosystems, CA, USA).

### NanoString Analysis

We employed NanoString analysis using custom nCounter XT probe. We hybridized 50 to 100 ng of RNA and then processed them with NanoString prep station and nCounter (NanoString Technologies). Differential gene expression levels were analyzed by NanoString nSolver software and normalized with expression of housekeeping genes.

### Immunofluorescence

Differentiated cells derived from hESCs were washed two times with 1XPBS and fixed in 4% paraformaldehyde in 0.1 M phosphate buffer saline (pH 7.4) (Santa Cruz Biotechnology, USA) for 20 min. The cells were permeabilized for 15 min with 0.5% Triton X-100 (Sigma, USA) in PBS (PBST), and blocked for at least 1 hour with 6% bovine serum albumin (BSA) in PBST at room temperature. Primary antibodies, including mouse anti-NKX6.1 (1:2000; DSHB; F55A12), guinea pig anti-PDX1 (1:1000; Abcam, Ab47308), and rat anti- Insulin (1:1000; GN-ID4, DSHB), goat anti-Glucagon (1:3000; sc-7781; SantaCruz) were added to the cells and incubated overnight at 4 °C. The next day, primary antibodies were removed, and 0.5% Tween 20 (TBST) was used for washing the cells 3 times. Subsequently, cells were incubated with the appropriate 488 and 568 Alexa Fluor secondary antibodies (Thermo Fisher Scientific) with a dilution of (1:500). Nuclear staining was examined after counterstain with Hoechst 33342 (1 μg/ml) (Thermo Fisher Scientific). The cells were visualized by Olympus IX53 inverted fluorescence microscope (Japan).

### Flow Cytometry

Differentiated hPSC-MPCs and hPSC-β cells were dissociated using TrypLE (Thermo Fisher Scientific, USA). Cells were fixed with 4% paraformaldehyde at 4 °C for 1 hour or overnight. They were later washed three times in PBS containing 0.2% bovine serum albumin (BSA) (Sigma, USA) and 0.1% saponin (Sigma, USA), blocked for 30 min at 4 °C in PBS containing 5% donkey serum and 0.1% saponin and then they were incubated with primary antibodies overnight at 4 °C. The primary antibodies were mouse anti-NKX6.1 (1:100; DSHB), guinea pig anti-PDX1 (1:100; Abcam), rabbit anti-Chromogranin A (CHGA) (1:1000; Abcam), goat anti-SOX9 (1:300; R&D Systems) and mouse anti- C-Peptide (1:100; Abcam), goat anti-Glucagon (1:250; SantaCruz). Cells were washed with PBS containing 0.2% BSA and 0.1% saponin and incubated with secondary antibodies in PBS for 1 h at room temperature. Stained cells were filtered through a 40-μm nylon mesh into flow cytometry tubes (BD Falcon) and were analyzed with BD Accuri™ C6 flow cytometer (BD Biosciences, USA) and FlowJo for data analysis.

### Plasmid Transfection, Cloning, Lentivirus Generation and Cells Transduction

For NKX6.1 overexpression, we used LacZpLX304 (addgene) and pMXs-NKX6.1-pLenti (addgene) for transfecting hPSCs during stage 4 differentiation. Cells were transfected using Lipofectamine STEM cat# (STEM00008) (Thermo Fihser Scientific). Cells were collected for analysis 48 hours post-transfection.

### Western Blotting

The protein concentrations were measured using the BCA Protein Assay kit (Thermo Fisher Scientific). Protein lysate was separated by gel electrophoresis on 10% SDS-polyacrylamide gels. Proteins were transferred to a PVDF membrane. The membranes were blocked for 1 hour at room temperature with 10% blotting-grade blocker (BioRad) in Tris-buffered saline (10 mM Tris, 150 mM NaCl, 0.1% Tween-20 pH 7.5) (TBST) followed by incubation with mouse anti-NKX6.1 (1:5000; DSHB) diluted in blocking buffer, overnight at 4 °C. After intensive washing (minimum 3 times) with TBST (5 min each time), the membranes were incubated with HRP-conjugated secondary antibody (1:10,000 diluted in blocking buffer) for one hour at room temperature, followed by intensive washing with TBST. The protein bands were visualized by iBright Western Blot Imaging Systems (Thermo Fisher Scientific).

### Statistical Analysis

All the data generated in this study are from at least three independent experiments. We used two-tailed Student’s t tests. A p value less than 0.05 was considered statistically significant.

## Results

### p53 Inhibition during Pancreatic Progenitor Stage Differentiation Enhances PDX1/NKX6.1 Co-Expression

**Fig. 2:**
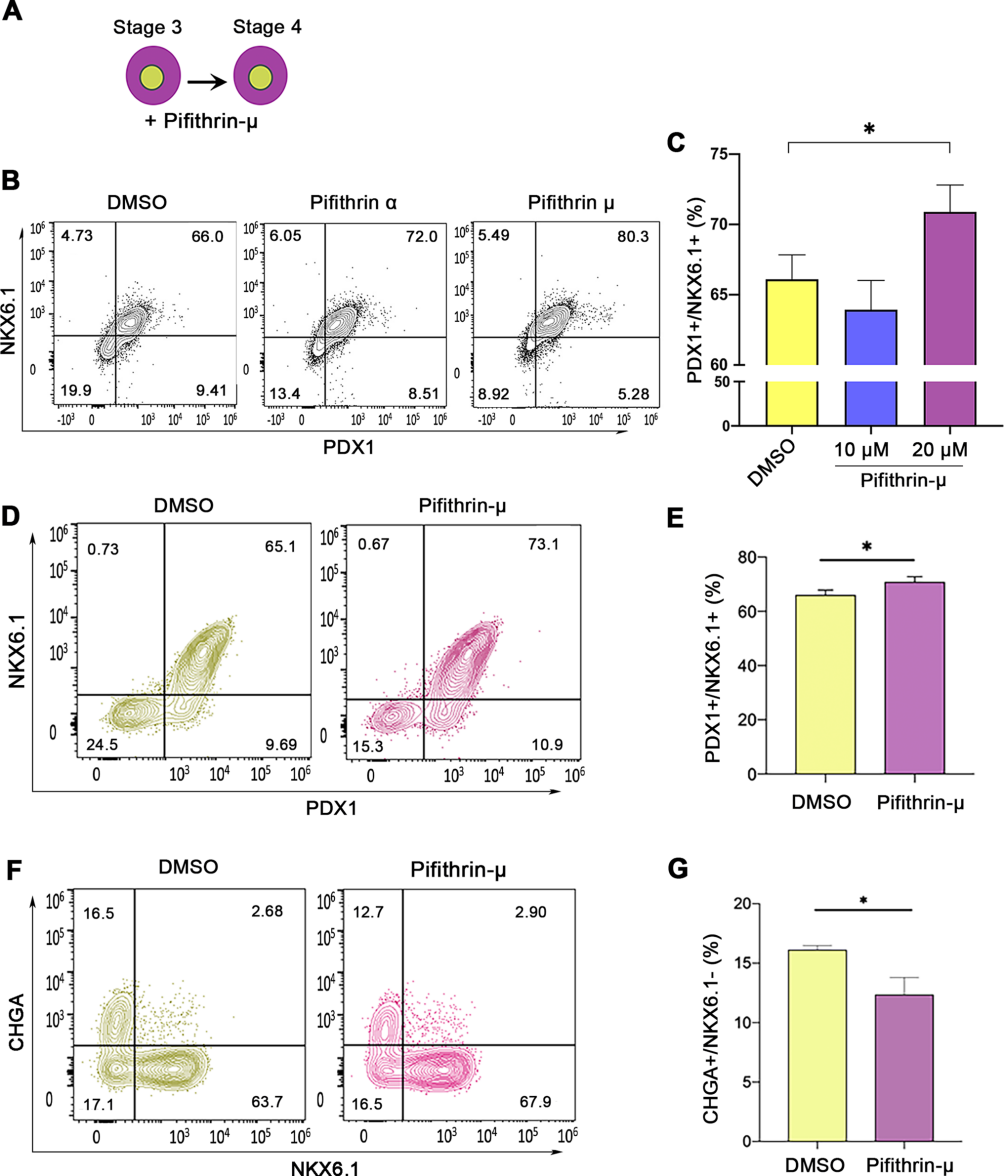
Inhibition of p53 enhances generation of hPSC-pancreatic progenitors. (**A**-**B**) Flow cytometry analysis and quantification showing the co-expression of PDX1 and NKX6.1 after treating the cells during stage 4 with DMSO, pifithrin-α, and pifithrin-μ. (**C**) Flow cytometry quantification of the co-expression of PDX1 and NKX6.1 after treating the cells during stage 4 with DMSO, and pifithrin-μ (10 μM and 20 μM). Flow cytometry analysis (**D**) and quantification (**E**) of PDX1 and NKX6.1 expression in DMSO or pifithrin-μ - treated pancreatic progenitors (*n* = 3). Flow cytometry analysis (**F**) and quantification (**G**) of CHGA and NKX6.1 expression in DMSO or Pifithrin-μ - treated cells (*n* = 3). Data are represented as mean ± SD; *p < 0.05, **p < 0.01, ***p < 0.001

**Fig. 3:**
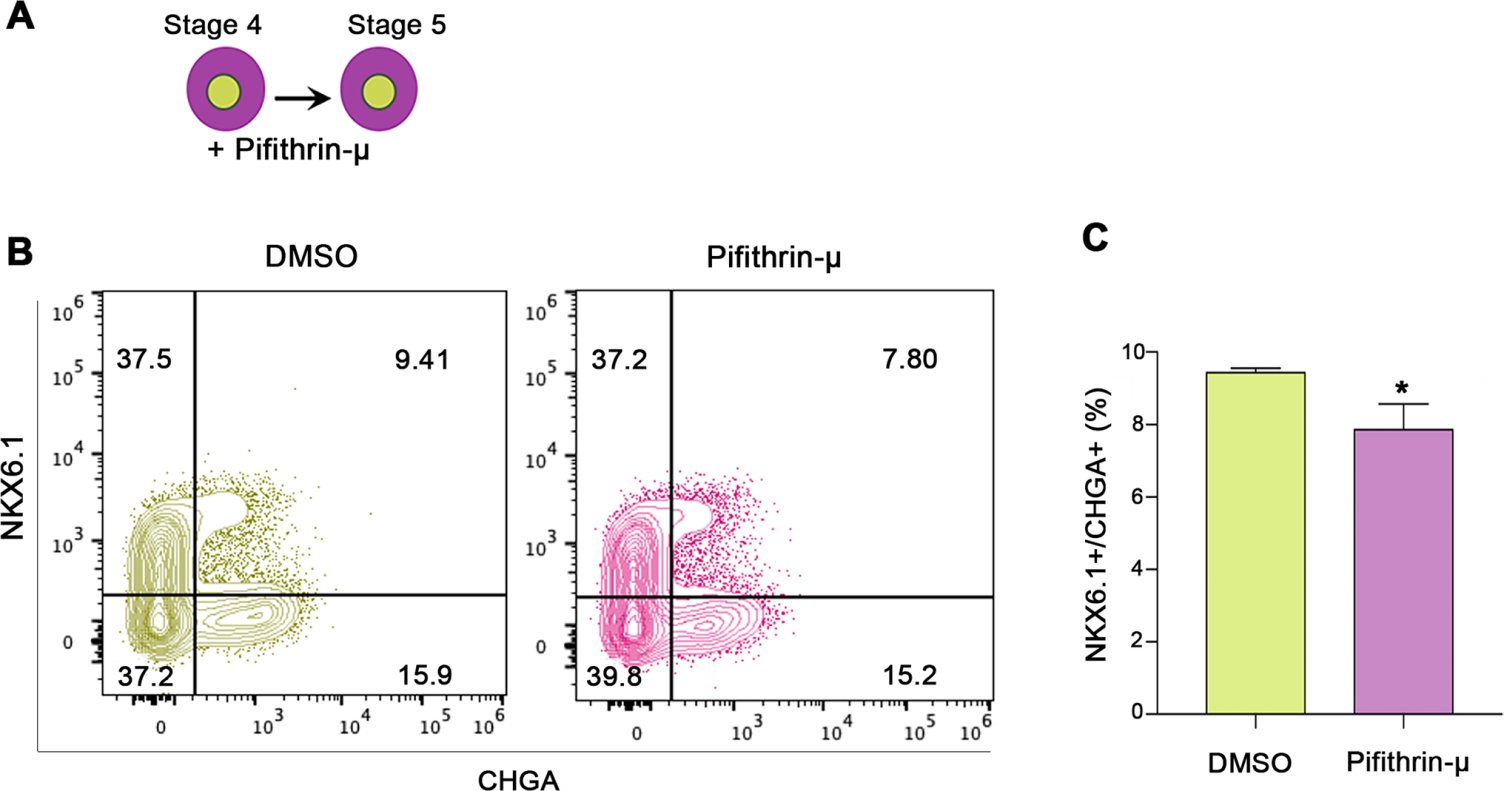
p53 activation is important for endocrine lineage commitment. (**A**) The cells were treated with DMSO and pifithrin-μ during stage 5 of differentiation. (**B**) Flow cytometry of CHGA and NKX6.1 expression in DMSO or pifithrin 𝜇 -treated. (**C**) Quantification of the proportion of CHGA+/NKX6.1+ expressing cells (*n* = 3). Data are represented as mean ± SD; *p < 0.05, **p < 0.01

hESCs were differentiated into pancreatic progenitors (MPCs) co-expressing PDX1 and NKX6.1 using our established protocol [20–22] (Fig. 1A, B). For further differentiation into pancreatic β-cells, Pagliuca et al. protocol was used [16] (Fig. 1B). To determine whether p53 inhibition would enhance the efficiency of MPCs by treating the cells during stage 4 of pancreatic β-cell differentiation with well-known p53 chemical inhibitors, pifithrin (PFT)-α and PFT-μ [23–25]. The cells were treated with PFT-α or PFT-μ for 4 days during stage 4 of differentiation (Fig. 2A). We found that both inhibitors increased the efficiency of MPCs co-expressing PDX1 and NKX6.1; however, the effect of PFT-μ was higher in comparison to PFT-α (Fig. 2B). Thus, we focused on using pifithrin-μ in all experiments. It is known that PFT-μ prevents the interaction between p53 and the mitochondrial transmembrane molecules Bcl-xL and Bcl-2 without hindering p53 transcriptional activities [25]. Furthermore, it is worth mentioning that PFT-μ effect on hPSC-derived MPCs was found to be dose-dependent. When we used a lower concentration (10 μM) of PFT-μ during the treatment of hPSC-MPCs, we noticed that there was no increase in the number PDX1^+^/NKX6.1^+^ expressing cells; however, increasing the concentration to 20 μM enhanced the generation of PDX1^+^/NKX6.1^+^ expressing cells (Fig. 2C). This indicates that 20 μM PFT-μ is an appropriate concentration that enhances MPC generation. There was a significant increase in the number of cells co-expressing the key pancreatic progenitor markers, PDX1 and NKX6.1, in the PFT-μ-treated cells compared to control cells treated with DMSO (Fig. 2D-E). Furthermore, the inhibition of p53 seemed to also help favor maintaining the cells in their progenitor’s state and prevent premature endocrine induction by reducing the differentiation of the cells into CHGA^+^/NKX6.1^−^ in the PFT-μ treated cells (Fig. 2F-G). Additionally, upon the addition of p53 inhibitor during stage 5 of differentiation, we noticed that there was a modest yet significant reduction on the number of CHGA^+^/NKX6.1^+^ cells, suggesting the role of p53 in specification to endocrine lineage (Fig. 3A-C). All in, these results suggest that p53 pathway has a role in the specification into MPCs and restraining the cells from early endocrine commitment.

### Inhibition of p53 during Pancreatic Progenitor Stage Favors Functional Monohormonal hPSC-β Cell Lineage

**Fig. 4:**
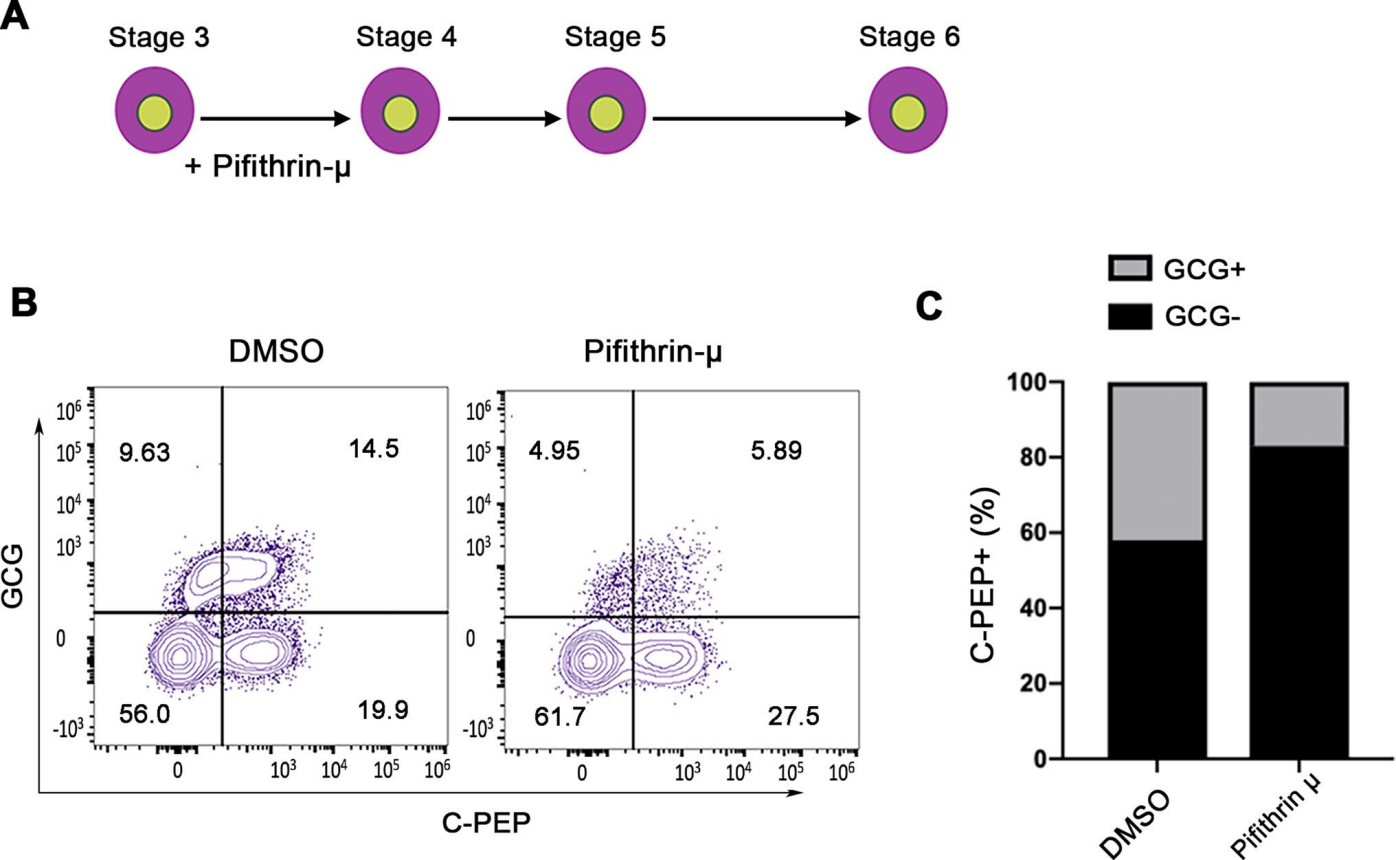
p53 inhibition enhances pancreatic progenitor differentiation into monohormonal β-cells. (**A**) Experimental design for B and C. Flow cytometry of C-peptide and Glucagon expression in DMSO or pifithrin 𝜇 -treated (**B**). Quantification of proportion of C- Peptide (C-PEP)/Glucagon (GCG) co-positive β-cells (**C**)

Next, we tested whether the above-mentioned increase in the efficiency of pancreatic progenitor’s specification attributed to p53 inhibition, can drive hPSC-MPC commitment to functional monohormonal hPSC-β cell fate. We carried out the differentiation of pifithrin-μ-treated hPSC-MPCs into pancreatic β-cells using Pagliuca et al. differentiation protocol [16]. Pifithrin-μ and DMSO-treated cells have generated the same number of cells expressing β-cell marker, C-Peptide (C-PEP) (Fig. 4A-C). However, our results showed that the number of cells co-expressing C-PEP and glucagon (GCG) (polyhormonal) was significantly reduced in the PFT-μ treated cells compared to controls (Fig. 4B-C). This indicates that the inhibition of p53 during stage 4 of differentiation has favored the differentiation of hPSC-MPCs into monohormonal hPSC-β cells instead of the hPSC-α cells lineage.

### Overexpression of NKX6.1 Suppresses p53 Expression and Enhances Pancreatic Progenitor Features

**Fig. 5:**
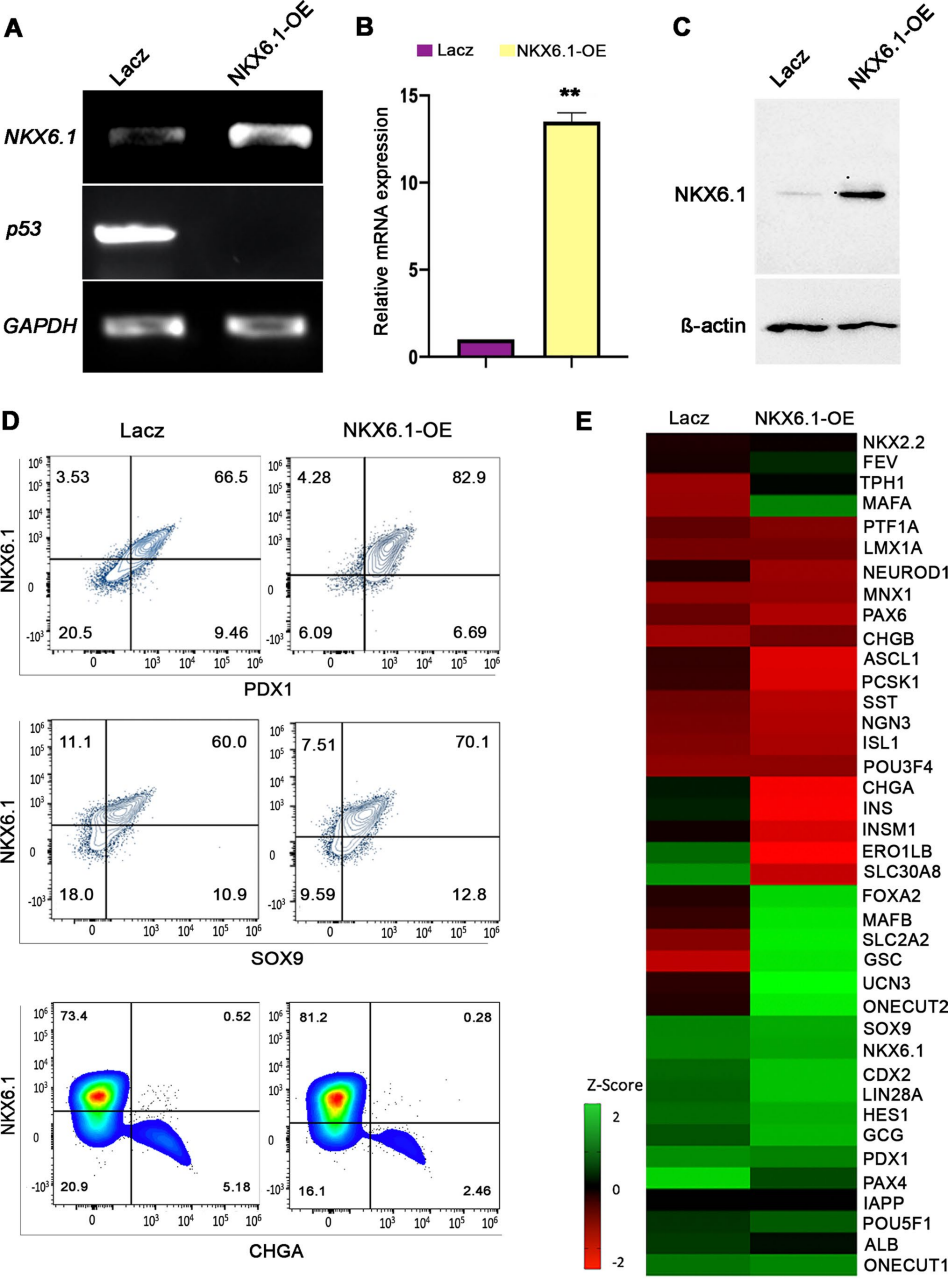
NKX6.1 overexpression in hPSC-derived pancreatic progenitors. (**A**) RT-PCR analysis showing the expression of *NKX6.1* in hESC-derived pancreatic progenitors, 48 hours after transfection with NKX6.1 and LacZ control*.* (**B**) RT-qPCR analysis of *NKX6.1* expression in hESC-derived pancreatic progenitors, 48 hours after transfection with NKX6.1 and LacZ control. (**C**) Western blot analysis showing the expression of NKX6.1, 48 hours after transfection with NKX6.1 and LacZ control. (**D**) Flow cytometry analysis of key pancreatic markers, NKX6.1, PDX1, SOX9, and CHGA, 48 hours after transduction of hESC-derived pancreatic progenitors with lentiviruses carrying lacZ control or NKX6.1. (**E**) Heatmap of Nanostring gene expression analysis comparing lacZ-transduced pancreatic progenitors compared to NKX6.1-transduced progenitors

To investigate the effect of NKX6.1 induction on p53 expression at pancreatic progenitor stage (stage 4), we overexpressed NKX6.1 during directed differentiation of hESCs into MPCs. Overexpression of NKX6.1 during hESC-MPCs differentiation was confirmed by an increase in its expression in comparison to control LacZ-transfected MPCs as examined by RT-PCR, qRT-PCR, and Western blotting (Fig. 5A-C). Interestingly, NKX6.1 overexpression dramatically suppressed p53 expression levels (Fig. 5A). Furthermore, as expected, NKX6.1 overexpression led to an increase in the number of PDX1^+^/NKX6.1^+^ MPCs (Fig. 5D), which is known as the source of functional pancreatic β-cells. We found that overexpression of NKX6.1 was accompanied by a marked increase in the expression of SOX9, which is one of the hallmarks of MPC stage, indicating the enhancement in the proportion of NKX6.1^+^/SOX9^+^ MPCs (Fig. 5D). Additionally, our flow cytometry data showed a decrease in the expression of the endocrine marker CHGA upon overexpressing NKX6.1 (Fig. 5D). Consistently, on mRNA level, we noticed that NKX6.1 overexpression resulted in a down-regulation of the endocrine markers, including *CHGA, NEUROD1*, *NGN3*, *INS*, and *SST* and upregulation of MPC markers, including *FOXA2*, *ONECUT1*, and *ONECUT2* at stage 4 of differentiation (Fig. 5E). This indicate that NKX6.1 overexpression prevents unsought immature endocrine induction and enhances MPCs induction. Furthermore, the expression of *PTF1A*, a marker that supports the specification of acinar cells [26], was notably decreased in NKX6.1 induced MPCs compared to control counterpart (Fig. 5E). Altogether, our results are consistent with the role of NKX6.1 in suppressing p53 expression and maintaining the main pancreatic progenitors’ features involved in the trajectory to monohormonal β-cells and blocking the route to undesirable production of immature polyhormonal endocrine cells.

## Discussion

Inadequate maturation of stem cell-derived pancreatic β-cells remains a significant hurdle in clinical translation due to their failure to secrete insulin in response to high glucose challenges. Several groups have introduced different small molecules to increase the efficiency of the differentiation protocols (Reviewed in [7, 27, 28]). This will ultimately lead to increasing the generation of more insulin-secreting β-cells. p53 is a transcription factor that plays different roles during development and in stem cells [29]. Previous studies reported conflicting results on the role of p53 in pancreatic β-cells. It has been demonstrated that p53 target genes are upregulated in diabetic mice [30] and p53 deletion does not change the insulin secretion or the number of β-cells in diabetes mouse models [31]. In contrast, another report showed that a specific deletion of p53 in mouse β-cells protects them against cell death induced by glucokinase mutation [32]. Furthermore, in mouse models of type 1 and type 2 diabetes, p53 inhibition maintains insulin secretion and glucose tolerance [33]. The inhibition of p53 expression has been found to be essential to maintain pancreatic progenitors during mouse development [34]. Although the same has not been reported in the human model, this notion showcased the role of p53 in the maintenance of pancreatic progenitors and their subsequent differentiation into endocrine cells. Therefore, in our study, we aimed to investigate the influence of p53 inhibition during pancreatic progenitors’ specification and how it will affect their commitment into pancreatic β-cells.

In the present study, we showed that using small molecules targeting p53 activity resulted in enhancing the differentiation of hESCs into MPCs co-expressing PDX1 and NKX6.1. The expression of these two markers is vital for driving the hPSCs-MPCs towards the functional monohormonal lineage [35]. The increase in the percentage of PDX1^+^/NKX6.1^+^ cells at the progenitor stage led to an increase in the number of monohormonal pancreatic β-cells. These findings establish the concept that the p53 inhibition during specification of MPCs is not only required for enhancing MPC generation but also, it’s critical for restricting them towards NKX6.1+ MPCs that give rise to functional β-cells. Therefore, we wanted to test if inducing the expression of NKX6.1 during MPCs differentiation will affect p53 expression. Interestingly, our data showed that the forced induction of NKX6.1 significantly diminished the expression of p53. It has been reported that inducing NKX6.1 expression in isolated primary rat islets results in induction of β-cell proliferation and aggravating their functionality by suppressing GCG expression and enhancing glucose-stimulated insulin secretion (GSIS) [36, 37]. However, no previous studies examined whether this defined role of NKX6.1 in β-cells can be extended to earlier stages of pancreas development such as pancreatic progenitor stage.

Our findings demonstrated that NKX6.1 overexpression upregulated key MPC markers crucial for β-cells lineage, such as SOX9, FOXA2, ONECUT1, and ONECUT2, which are known to be a critical TF to initiate subsequent endocrine specification (reviewed in [10]). Furthermore, previous studies have reported that polyhormonal hPSC-β-cells originate due to precocious immature endocrine induction in PDX1-expressing hPSC-MPCs that lacked NKX6.1 expression [15]. Consistently, our results showed that endocrine markers including *CHGA*, *INS*, and *SST* were suppressed upon inducing NKX6.1 in MPCs. This highly supports the notion that one of the ways NKX6.1 expression promotes the formation of functional β-cells is by preventing unsought induction of endocrine differentiation [15]. Additionally, we showed that the forced expression of NKX6.1 abrogated non-endocrine lineage marker *PTF1A*, which is accountable for acinar cells commitment. All in, this strongly proposes that NKX6.1 expression at the MPC stage is crucial for pancreatic β-cell specification through inhibiting p53 expression.

Our study demonstrated for the first time that regulating p53 expression with the use of effective chemical inhibitors during the directed differentiation into hPSC-β cells may improve their efficiency and subsequently their functionality. In our study, we further differentiated the MPCs treated with p53 inhibitor (PFT-μ) towards the β-cell lineage. While the number of C-PEP-expressing cells was similar between the two groups, the number of the polyhormonal cells co-expressing C-PEP and GCG (α-cell marker) was markedly decreased in the PFT-μ -treated group compared to control DMSO-treated cells. The difference in the ratios between the generated α- and β-cells is crucial to acquire improved insulin secretion and subsequently enhance their functionality. This finding can be potentially used to optimize existing differentiation protocols with the addition of p53 inhibitor during MPC specification to enhance the functionality of the generated β-cells. Previous reports suggested that suppressing p53 expression may induce genome instability and tumor formation [38]. However, in our study, the small molecule was used transiently during the pancreatic progenitor stage (4 days) only. This stage precedes the endocrine progenitor specification (stage 5) and mature β-cells stage (stage 6), suggesting that the generated β-cells express normal p53 level. Therefore, we believe that the transient addition of the p53 inhibitor during the MPC differentiation will not lead to genetically altered β cells as opposed to long-term treatment or permanent deletion of p53. Additionally, in cellular therapy, hPSC-derived β-cells are undergone extensive screening, including the evaluation of the genetic stability of the generated cells, for safety prior to transplantation. Nevertheless, further studies aiming to dissect the exact role of p53 during the different stages of pancreatic β-cell development is still needed.

The role of p53 in the stem cell maintenance and differentiation into other lineages has been previously reported. For instances, p53 has been found to play essential role in maintaining ESC self-renewal under different conditions [39–41]. Several studies reported that p53 inhibition enhances differentiation of mesenchymal stem cells (MSCs) into bone cells [42–44]. Other studies demonstrated that suppression of p53 accelerates neural differentiation from neuroblasts [45] and ESCs [46] and inhibits the differentiation of ESCs into adipocytes and smooth muscle cells [46]. Another group of scientists showed that the inactivation of p53 is necessary to initiate epidermal differentiation in human keratinocytes [47]. Moreover, p53 silenced cells have been used for transplantation when Kundu and colleagues used p53-silenced endothelial progenitor cells for treating diabetic kidney disease in a mouse model [48]. Other studies on cardiovascular cells showed that ectopic expression of miR-27b in diabetic bone marrow-derived progenitor cells (BMPCs) of the endothelium reduces p53 expression [49], and suppression of histone deacetylase 1 (HDAC1) enhances cardiac and endothelial lineage markers in human cardiac-derived mesenchymal stromal cells (CMCs) by inducing the expression of p53 [50]. Altogether, this showcases the importance of p53 manipulation at precise time points during differentiation of other cell types.

## Conclusion

Overall, our findings have described a novel role for p53 in the regulation of pancreatic progenitor maintenance in human. Furthermore, we showed that p53 inhibition during hPSC-MPC differentiation is a critical determinant of specification between pancreatic α- and β-cell lineages. p53 expression at pancreatic progenitor stage and endocrine progenitors stage exhibited a dichotomous role in these two opposing endocrine lineages. Nevertheless, the defined mechanism which by p53 plays this crucial role needs further studies.

## Supplementary Information

ESM 1 (DOCX 15 kb)

ESM 2 (DOCX 13 kb)

## Data Availability

The data that support the findings of this study are available from the corresponding author upon reasonable request.
