# Supplementary material for: p53 Inhibition in Pancreatic Progenitors Enhances the Differentiation of Human Pluripotent Stem Cells into Pancreatic β-Cells
Source: Stem Cell Rev Rep. 2023 Jan 28;19(4):942–52. doi: 10.1007/s12015-023-10509-1 (PMC10185607; doi:10.1007/s12015-023-10509-1)
Supplement: Supplementary file 1 — (DOCX 15 kb) [file 12015_2023_10509_MOESM1_ESM.docx]

**Supplementary Table 1:** Differentiation of hESCs into pancreatic progenitors and pancreatic β-cells

| **Stages** | **Reagents** |
| --- | --- |
| **Stage 1**  **(4 days)** | **Day 1:** MCD131 media was supplemented with 100 ng/mL Activin A (R&D Systems, USA), 2 μM CHIR99021 (Stemgent, USA), 10 μM Y-27632 (Stemgent, USA) and 0.5 mM Vitamin C (Sigma, USA).  **Days 2-4:** MCD131 media with 100 ng/mL Activin A and 0.5 mM Vitamin C. |
| **Stage 2**  **(2 days)** | **Days 1-2:** DMEM/F12 with 200 nM LDN193189, 50 ng/mL FGF10, 0.5 mM Vitamin, 2 μM RA (Sigma, USA), 0.25 mM SANT1 (Sigma, USA), 1% of B27 (ThermoFisher Scientific, USA) and 0.5 mM Vitamin C. |
| **Stage 3**  **(2 days)** | **Days 1-2:** DMEM/F12 with 200 nM LDN193189, 50 ng/mL FGF10, 0.5 mM Vitamin, 2 μM RA (Sigma, USA), 0.25 mM SANT1 (Sigma, USA), 1% of B27 (ThermoFisher Scientific, USA) and 0.5 mM Vitamin C. |
| **Stage 4**  **(4 days)** | DMEM/F12 with 200 nM LDN193189, 100 ng/mL EGF, 10 mM Nicotinamide (Sigma, USA), 1% of B27 and 0.5 mM Vitamin C.  Cells were collected and analyzed at the end of stage 4. |
| **Stage 5**  **(7 days)** | **Days 1-4:** MCDB131 was supplemented with 0.25 μM Sant1, 1 μM γ secretase (EMD Millipore), 100 nM RA, 10 μM Alk5i II (Axxora), 20 ng/mL Betacellulin (Thermo Fisher Scientific), 1 μM T3 (EMD Millipore).  **Days 5 and 7:** Media was supplemented with: 25 nM RA, 1 μM γ secretase, 10 μM Alk5i II, 20 ng/mL Betacellulin and 1 μM T3. |
| **Stage 6**  **(14 days)** | **Days 1–14** (media change every other day) with MCD131 media.  Cells were analyzed on day 14th of the protocol. |
