# Supplementary material for: p53 Inhibition in Pancreatic Progenitors Enhances the Differentiation of Human Pluripotent Stem Cells into Pancreatic β-Cells
Source: Stem Cell Rev Rep. 2023 Jan 28;19(4):942–52. doi: 10.1007/s12015-023-10509-1 (PMC10185607; doi:10.1007/s12015-023-10509-1)
Supplement: Supplementary file 2 — (DOCX 13 kb) [file 12015_2023_10509_MOESM2_ESM.docx]

**Supplementary Table 2.** The list of primer sequences used for RT-PCR and RT-qPCR analyses

| **Gene** | **Sequence** | **Product size** | **Cycle number for PCR** |
| --- | --- | --- | --- |
| ***GAPDH*** | F: ACGACCACTTTGTCAAGCTCATTTC | 132 | 25 |
|  | R: GCAGTGAGGGTCTCTCTCTTCCTCT |  |  |
|  | R: CCGTGAGATGTACTTGTTGAATAGGA |  |  |
| ***NKX6.1*** | F: CTTCTGGCCCGGAGTGATG | 114 | 35 |
|  | R: GAAGAGAAAACACACGAGACCC |  |  |
| ***p53*** | F: GTCCCAAGCAATGGATGATTTG | 589 | 32 |
|  | R: GTTGTAGTGGATGGTGGTACAG |  |  |
